# Filamentous tangles with nemaline rods in *MYH2* myopathy: a novel phenotype

**DOI:** 10.1186/s40478-021-01168-9

**Published:** 2021-04-29

**Authors:** Nicolas N. Madigan, Michael J. Polzin, Gaofeng Cui, Teerin Liewluck, Mohammad H. Alsharabati, Christopher J. Klein, Anthony J. Windebank, Georges Mer, Margherita Milone

**Affiliations:** 1 Department of Neurology, Mayo Clinic, 200 1st St SW, Rochester, MN 55905 USA; 2 Department of Biochemistry and Molecular Biology, Mayo Clinic, 200 1st St SW, Rochester, MN 55905 USA; 3 Department of Neurology, UnityPoint Health, 1221 Pleasant St Suite 300, Des Moines, IA 50309 USA

**Keywords:** Congenital myopathy, *MYH2*, MyHC-IIA, Myosin heavy chain IIA, Nemaline rods, Sarcomeric protein aggregation, Ophthalmoplegia, Rimmed vacuoles, Type 2A fiber atrophy, Type 2A fiber loss

## Abstract

The *MYH2* gene encodes the skeletal muscle myosin heavy chain IIA (MyHC-IIA) isoform, which is expressed in the fast twitch type 2A fibers. Autosomal dominant or recessive pathogenic variants in *MYH2* lead to congenital myopathy clinically featured by ophthalmoparesis and predominantly proximal weakness. *MYH2*-myopathy is pathologically characterized by loss and atrophy of type 2A fibers. Additional myopathological abnormalities have included rimmed vacuoles containing small p62 positive inclusions, 15–20 nm tubulofilaments, minicores and dystrophic changes. We report an adult patient with late-pediatric onset *MYH2*-myopathy caused by two heterozygous pathogenic variants: c.3331C>T, p.Gln1111* predicted to result in truncation of the proximal tail region of MyHC-IIA, and c.1546T>G, p.Phe516Val, affecting a highly conserved amino acid within the highly conserved catalytic motor head relay loop. This missense variant is predicted to result in a less compact loop domain and in turn could affect the protein affinity state. The patient’s genotype is accompanied by a novel myopathological phenotype characterized by centralized large myofilamentous tangles associated with clusters of nemaline rods, and ring fibers, in addition to the previously reported rimmed vacuoles, paucity and atrophy of type 2A fibers. Electron microscopy demonstrated wide areas of disorganized myofibrils which were oriented in various planes of direction and entrapped multiple nemaline rods, as corresponding to the large tangles with rods seen on light microscopy. Nemaline rods were rarely observed also in nuclei. We speculate that the mutated MyHC-IIA may influence myofibril disorganization. While nemaline rods have been described in myopathies caused by pathogenic variants in genes encoding several sarcomeric proteins, to our knowledge, nemaline rods have not been previously described in *MYH2*-myopathy.

## Introduction

Myosins are mechano-enzymes which hydrolyze adenosine triphosphate (ATP) and interact between actin and M-band sarcomeric filaments in muscle to generate force and movement [11]. The molecular power stroke cycle, as the fundamental basis for muscle movement, is the result of changes to myosin heavy chain (MyHC) affinity with actin upon ATP binding, hydrolysis and release [7], and may occur up to 300 times per second depending on the contractile demand [8]. Pathologic variants in myosin heavy chain genes therefore may profoundly affect muscle function and structure. Hundreds of mutations have been identified in the *MYH*7 gene [7], which encodes the beta heavy chain of myosin of type I skeletal muscle fibers and cardiac muscle. Pathologic *MYH7* variants lead to skeletal myopathies [4, 20, 37] sometimes accompanied by myosin storage, and to cardiomyopathy [28].

Far fewer mutations (Table 1), inherited as autosomal dominant or recessive traits, have been reported in the *MYH*2 gene, encoding the MyHC-IIA isoform of fast-twitch type 2A muscle fibers [3, 5, 14, 15, 25, 29, 36]. Regardless of the genotype and pattern of inheritance, *MYH2*-myopathy often manifests also with ophthalmoparesis, as a likely consequence of the high proportion of type 2A muscle fibers within extraocular muscles [1, 2, 10, 31]. Autosomal recessive phenotypes tend to be milder than dominant forms and to lack early contractures, but both have early disease onset [12, 13, 26, 27]. Muscle histopathology shows selective paucity or absence of type 2A fibers, accompanied by minimal abnormalities [36], dystrophic changes [28], minicores [28], intranuclear and cytoplasmic inclusions or rimmed vacuoles [29]. Rimmed vacuoles and inclusions were initially reported in autosomal dominant *MYH2*-myopathy. Both were later detected also in the recessive form [29], sometimes in combination with ultrastructural evidence of disarrangement of the myofilaments [28], suggesting a spectrum of pathological findings independently from the modality of inheritance.

**Table 1:** MyHC-IIA variants by location and clinical features
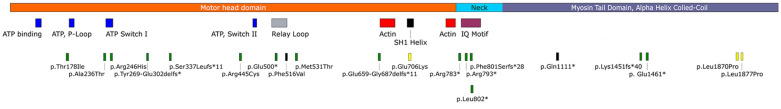

Inheritance	Pathogenic variant	Subdomain	Clinical features of pronounced ophthalmoparesis, with	References
*Motor head and neck*				
Autosomal Dominant (yellow bar)	c.2166G>A, p.Glu706Lys	SH1 helix	Classical form: joint contractures in infancy (outgrown); progressive proximal limb muscle weakness; hand weakness and tremor; mild facial weakness; axial hyperlordosis and kyphoscoliosis	[3, 14, 15, 29]
Autosomal recessive (green bar)				
Homozygous	c.1009-1G>A, p.Ser337Leufs*11	Exon 10 skipping	Dysphagia in infancy; ptosis; tall, thin, with scoliosis; mild truncal and proximal limb muscle weakness	[36]
Homozygous	c.737 G>A p.Arg246His	ATP-binding, Switch I	Proximal and facial muscle weakness, ptosis, dysphagia, joint laxity	[5]
Homozygous	c.2400delG, p.Phe801Serfs*28	IQ motif	Facial, neck flexor, upper and proximal lower limb muscle weakness; scoliosis	[12, 13]
Homozygous	c.2398delG, p.Gly800fs27*	IQ motif	Severe dysphagia in infancy, neck and proximal muscle weakness	[31]
Heterozygous	c.904+1G>A, p.Tyr269-Glu302delfs* c.2347C > T p. Arg783*	Exon 9 Actin binding—IQ motif	Facial, neck, elbow and ankle flexion; mild proximal muscle weakness; ptosis; joint hypermobility	[25]
Heterozygous	c.1975-2A>G, p.Glu659-Gly687delfs*11 c.2405T>A p.Leu802*	Actin binding IQ motif	Facial, upper and proximal lower limb, and abdominal muscle weakness; congenital pectus carinatum	[27]
Heterozygous	c.1331C>T, p.Arg445Cys c.2405T>A, p.Leu802*	Exon 12 IQ motif	Early childhood onset; facial, neck flexor, proximal arm, hand, hip flexor and abdominal muscle weakness	[26]
Homozygous	c.533C>T, p.Thr178Ile	ATP binding, P-loop	Early childhood onset; facial, neck flexor, proximal limb and hand muscle weakness; lumbar lordosis	[26]
Homozygous	c.706G>A, p.Ala236Thr	ATP-binding, Switch I	Early childhood onset; facial, neck flexor, proximal limb and hand muscle weakness	[26]
Homozygous	c.1592T>C, p.Met531Thr	Exon 14	Adolescent onset; facial, neck flexor, proximal limb and hand muscle weakness; finger contractures	[26]
Homozygous	c.1498G>T, p.Glu500*	Relay loop	Neck flexor, proximal upper and lower limb muscle weakness; early cataracts	[6]
*Coiled-coil tail*				
Autosomal dominant (yellow bar)	c.5609T>C, p.Leu1870Pro	Helix heptad disruption, ‘d’ position	Neonatal onset; severe dysphagia; facial muscle weakness and ptosis; scoliosis; waddling-steppage gait	[2]
Autosomal dominant (yellow bar)	c.5630T>C p.Leu1877Pro	Helix heptad disruption, ‘d’ position	Dysphagia; prominent distal > proximal muscle weakness; ptosis; asymetrical scapular winging	[1]
Autosomal recessive (green bar)				
Homozygous	c.4352delA, p.Lys1451Serfs*40	Exon 29, tail truncation	Neonatal onset; facial, neck flexor, and proximal limb weakness; marked ptosis	[26]
*Motor head+coiled coil tail*				
Autosomal Recessive				
Heterozygous (green bar)	c.2377C>T, p.Arg793*	IQ motif, truncation	Proximal lower, then upper limb muscle weakness; scapular winging; ptosis and diplopia; waddling gait	[30]
	c.4381G>T, p.Glu1461*	Tail truncation		
Heterozygous (black bar)	c.1546T>G, p.Phe516Val	Relay loop	Upper limb and proximal lower limb muscle weakness; facial weakness and ptosis; joint laxity; waddling gait	This report
	c.3331C>T, p.Gln1111*	Tail truncation		

In this study, our aim is to highlight the novel myopathological findings of a patient with *MYH2*-myopathy culminating in MyHC-IIA protein expression loss, filamentous tangles and clusters of nemaline rods.

## Case presentation

### Clinical, serological and electrophysiological findings

**Fig. 1:**
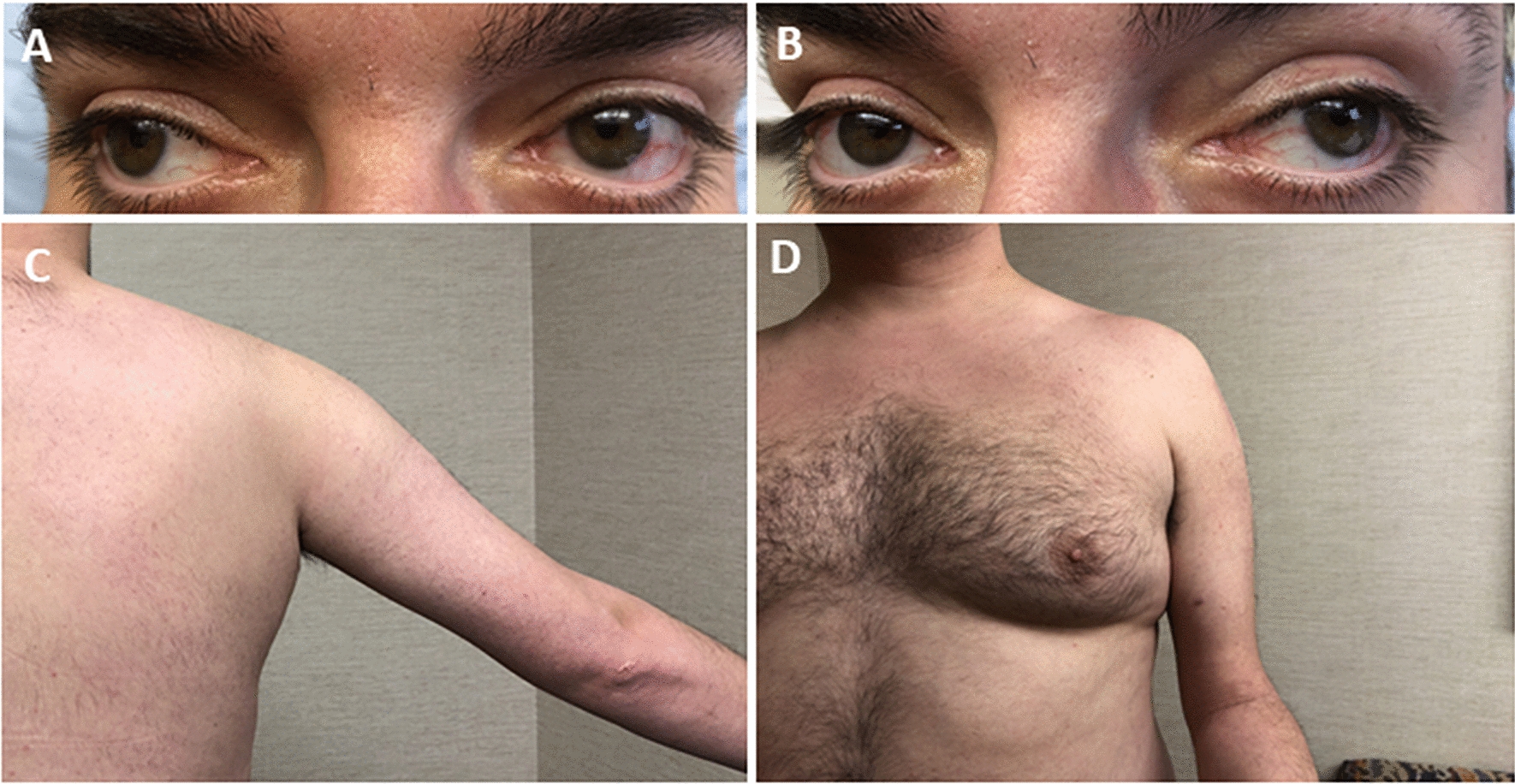
Clinical features. Patient’s photographs demonstrating (**a**, **b**) bilateral ophthalmoparesis limiting his lateral gaze bilaterally, (**c, d**) shoulder and arm muscle atrophy and (**d**) gynecomastia

A 34 year old man of English and Irish descent was born from a normal pregnancy and had normal motor development. He had no history of early joint contractures. He was notably tall and thin in elementary school. In middle school, he started experiencing difficulty keeping up with peers in sprinting and jumping activities. Limitations to his range of eye movements were first noticed by peers at age 23. By age 26, he had difficulty climbing stairs, while a year prior to his clinical presentation, he developed mild shoulder muscle weakness in the absence of myalgia. He had no dysphagia or dyspnea. His parents and two sisters were unaffected by muscle weakness (father was deceased but had no history of weakness). Neurological examination was notable for mild bilateral ptosis and ophthalmoparesis (Fig. 1a, b), mild weakness of facial and neck flexor muscles 4+/5, as graded by the Medical Research Council (MRC) scale, symmetric muscle atrophy and weakness of the shoulder girdle muscles (Fig. 1c, d), brachioradialis and finger extensors (4, 4+/5), severe weakness of hip flexors (3/5 bilaterally), less severe involvement of thigh adductors and abductors, and ankle dorsiflexors (4+/5). The quadriceps were symmetrically atrophic but with spared strength. Axial weakness was suggested by patient’s inability to sit up from a supine position without assistance. He demonstrated a waddling gait and was able to walk on toes but not on heels. Tendon reflexes and sensory examination were normal. He had a high arched palate, laxity of the metacarpal phalangeal joints and mild gynecomastia (Fig. 1d), but no contractures. No tremor was present.

Creatine kinase (CK) levels were elevated (457–626 U/L; normal < 320). The electrocardiogram and echocardiography were normal. He had a reduced maximal expiratory pressure (73% of predicted) and normal vital capacity on pulmonary function testing. Concentric needle electromyography demonstrated mixed small and large motor unit potentials in proximal limb and thoracic paraspinal muscles with early recruitment, fibrillation potentials in the gastrocnemius and complex repetitive discharges in proximal lower limb muscles. Nerve conduction studies were normal. 2-Hz repetitive stimulation of the facial, spinal accessory and peroneal nerves showed no decrement. (Limited information on this patient were reported prior to the pathological characterization of the myopathy [17]).

### Muscle biopsy findings

**Fig. 2:**
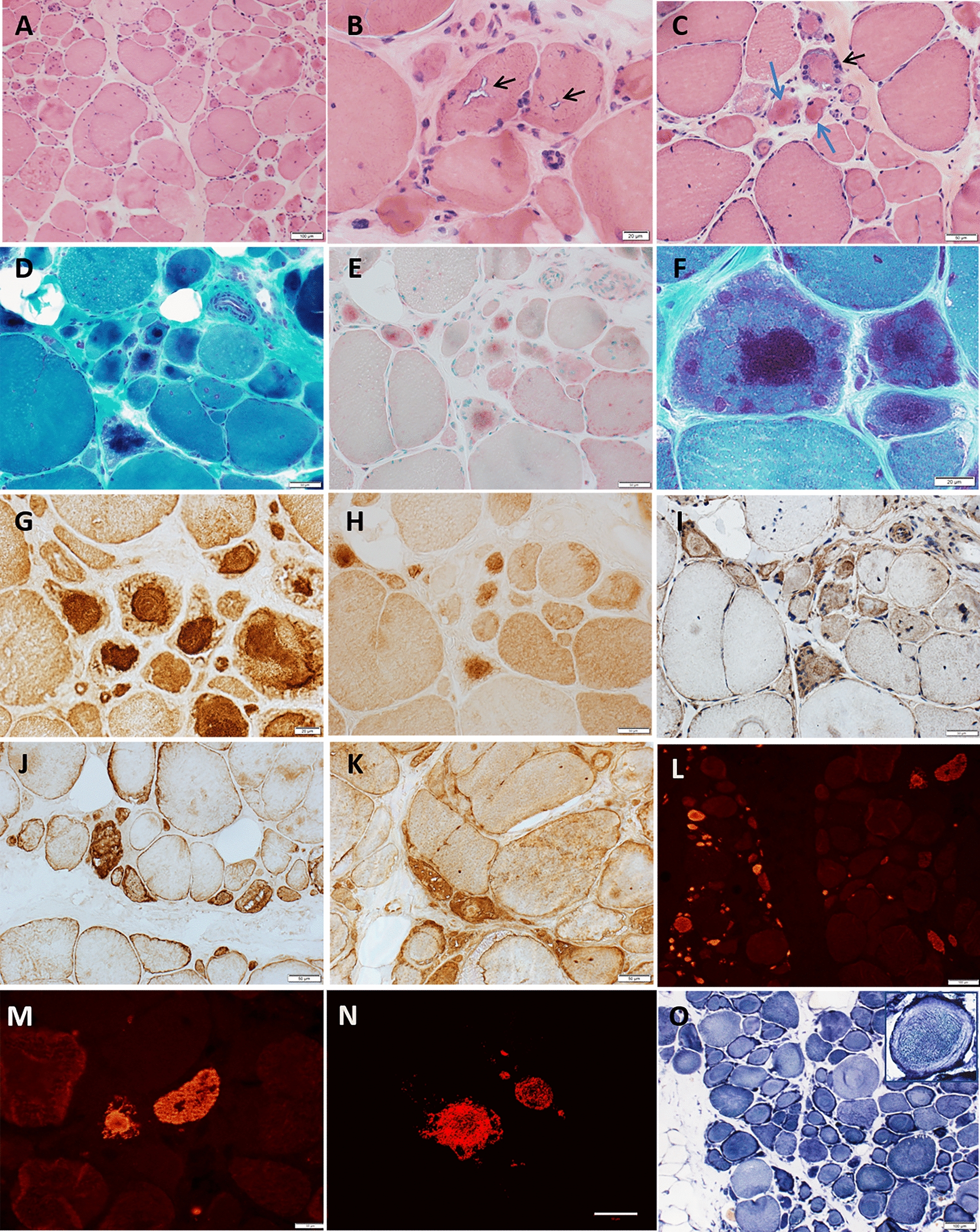
Patient’s muscle biopsy. **a–c** Hematoxylin and eosin staining demonstrating a chronic myopathic process with wide-ranging muscle fiber size variability, fiber splitting, internalized nuclei, and increases in endomysial fatty connective tissue. **b** Rimmed vacuoles are seen in occasional muscle fibers (black arrows). **c** Atrophic fibers showing large subsarcolemmal nuclei (black arrow) or eosinophilic inclusions (blue arrows), which stain dark red (**d**) in trichrome and red (**e**) in acid phosphatase. **f** A higher magnification trichrome stained section shows that the large dark red central inclusions have a filamentous appearance and tiny rod-like structures. **g** Such filamentous tangles contain abundant alpha-actinin and **h** less myotilin, but not **i** p62, **j** desmin or **k** alphaB-crystallin, all of which are diffusely and often faintly increased (light brown) in some atrophic fibers. **l** MyHC-IIA immunohistochemical staining demonstrates paucity and atrophy of type 2A fibers, which harbor (**m**) filamentous tangles containing MyHC-IIA, as also demonstrated by (**n**) Z-stacked confocal microscopy in an immediately adjacent muscle section (bar = 50 microns). **o** NADH dehydrogenase reacted section showing numerous ring fibers (one is shown in the right upper corner at higher magnification, 40×) and overreactive (dark blue) atrophic fibers. Primary mouse anti-human MyHC-IIA monoclonal antibody [9, 35] (A4.74, 1:100 dilution, Developmental Studies Hybridoma Bank (DSHB) (University of Iowa, Iowa City, Iowa, USA) was visualized with Cy3-conjugated affinity purified donkey anti-mouse IgG (1:200, Millipore Chemicon, Temecula, California USA)

Routine histological studies of the triceps biopsy demonstrated wide fiber size variability, muscle fiber splitting (Fig. 2a), internalized nuclei, rare necrotic or regenerating fibers and occasional rimmed vacuoles (Fig. 2b). The perimysial and endomysial connective tissue were increased suggesting chronicity. Several fibers, mainly atrophic fibers, contained prominent subsarcolemmal nuclei and/or large (up to 30 microns in diameter) central accumulation of eosinophilic material (Fig. 2c) that stained dark red in trichrome (Fig. 2d) and overreacted for acid phosphatase (Fig. 2e). At a higher magnification (Fig. 2f) these large central inclusions had the appearance of a filamentous tangle or a “ball of yarn” and often contained nemaline rods of various size. Immunohistochemical studies (performed as previously reported [22]) demonstrated that these tangles overreact for sarcomeric alpha-actinin and to a lesser degree for myotilin (Fig. 2g, h), but not for sequestome 1 (p62), desmin, alphaB-crystallin (Fig. 2i–k) or neural cell adhesion molecule 1 (NCAM 1) (not shown), all of which were diffusely increased in fewer scattered atrophic fibers. The tangles did not overreact for dystrophin. Under rhodamine optics, only extremely rare atrophic fibers showed focal large congophilia and a single non-atrophic fiber demonstrated small subsarcolemmal congophilic inclusions (not shown). Immunohistochemical studies using an antibody against human MyHC-IIA protein (A4.74, Developmental Studies Hybridoma Bank (DSHB) (University of Iowa, Iowa City, Iowa, USA) [35] demonstrated paucity and atrophy of type 2A fibers (Fig. 2l), and MyHC-IIA positivity of the fibers harboring the filamentous tangles, as observed by optical (Fig. 2m) and confocal microscopy (Fig. 2n). Additionally, sections reacted for reduced nicotinamide adenine dinucleotide (NADH) dehydrogenase showed a myriad of ring fibers, lobulated fibers and several atrophic fibers that were diffusely overreactive (Fig. 2o). NADH enzyme reactivity was preserved or increased in correspondence of the tangles.

**Fig. 3:**
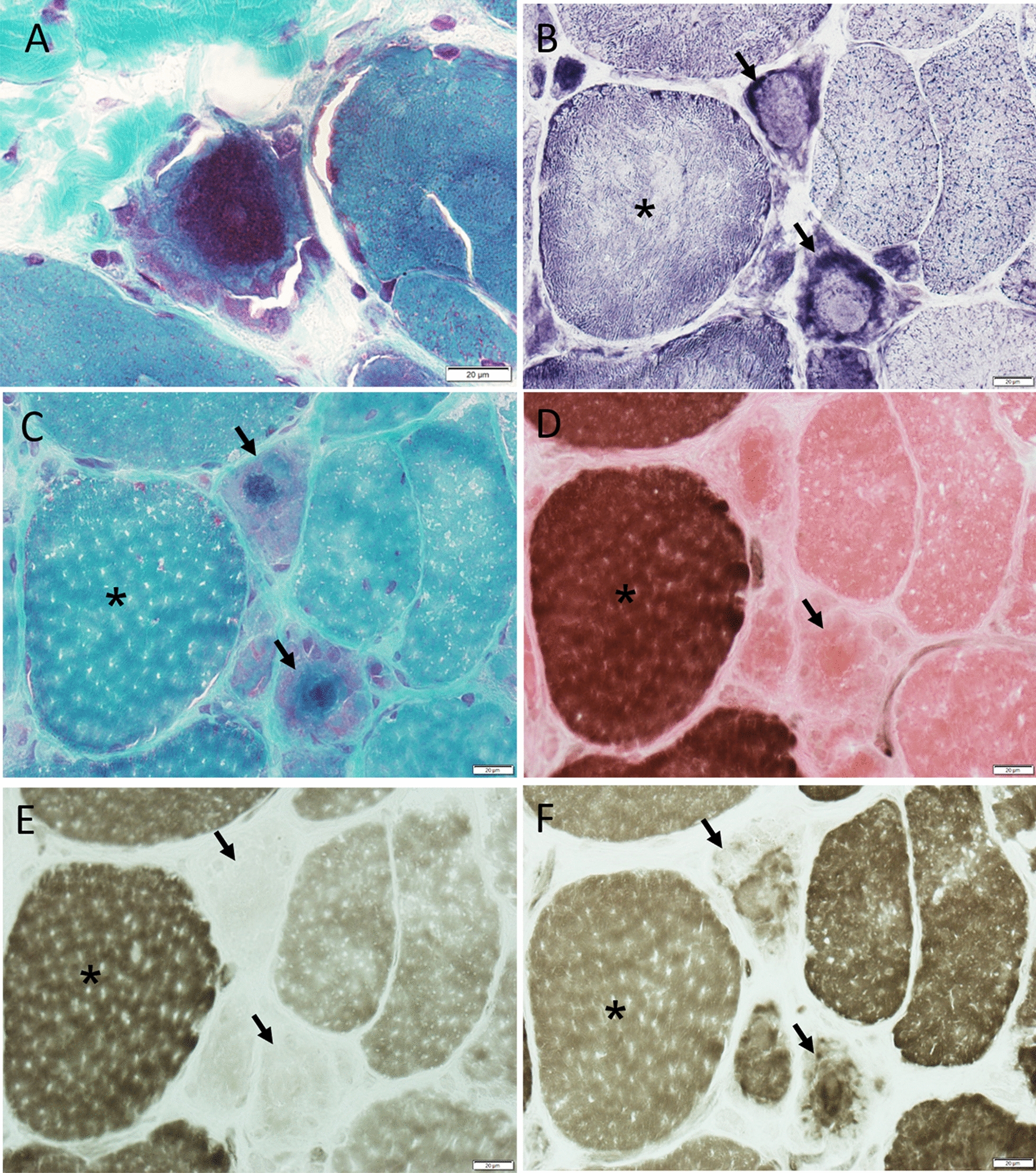
Additional images of patient’s muscle biopsy. **a** High magnification trichrome stained section showing the filamentous appearance of a large tangle and the associated nemaline rods (dark red). **b–f** Serial sections showing two fibers (arrows; the asterisk indicate the reference fiber) with preserved and increased NADH reactivity centrally and peripherally, respectively (**b**), harboring the tangles with nemaline rods (**c**, trichrome) and being type 2A in ATPase sections reacted at various pH (**d** ATPase 4.3; **e** ATPase 4.6; **f** ATPase 9.4)

**Fig. 4:**
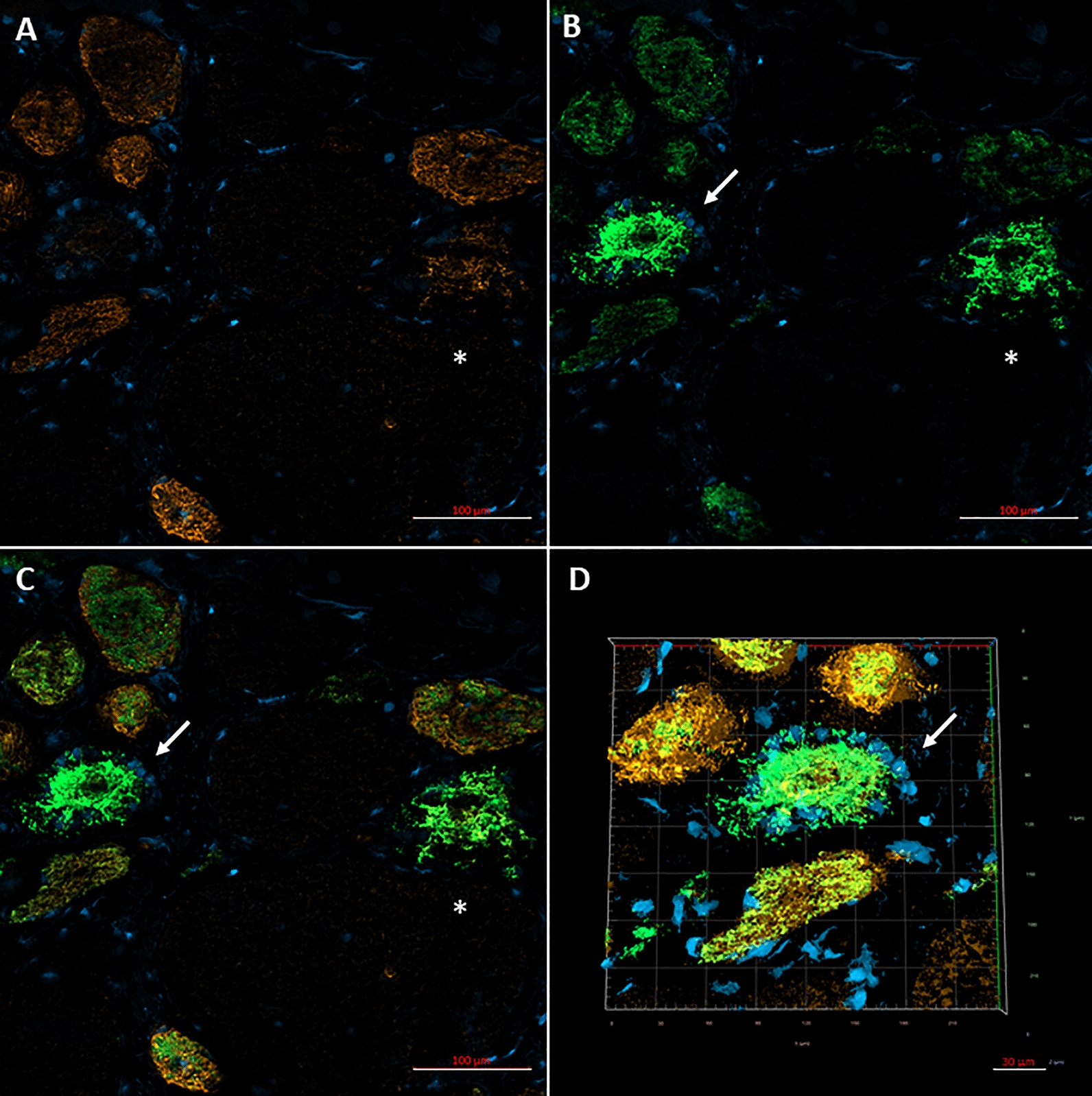
Immunofluorescence with confocal imaging identifies MyHC-IIX, MyHC-IIA,and IIA/IIX hybrid fibers. **a** Immunostaining for MyHC-IIX (orange) using the 6H1 antibody (mouse anti-human IgM, 1:1 dilution, Developmental Studies Hybridoma Bank (DSHB), and **b** for MyHC-IIA (green, intensely staining) and IIX proteins (weakly staining) (A4.74 antibody, 1:200 dilution) [16] are merged in **c** with DAPI nuclear staining. The series identifies a IIA fiber (arrows) and a hybrid IIA/IIX fiber (asterisk) that demonstrate filamentous changes and centralized myofiber disorganization. The IIX fibers display relatively normal size and architecture. **d** Confocal Z-stack imaging provides further detail of the 3-dimensional structure of the filamentous changes in the IIA fiber. Primary antibodies were visualized using Alexa Fluor® 488 conjugated goat anti-mouse IgG1 (A4.74) and Alexa Fluor® 555 conjugated goat anti-mouse IgM secondary antibodies at 1:200 dilutions (Invitrogen / ThermoFisher Scientific, Waltham, MA USA)

**Fig. 5:**
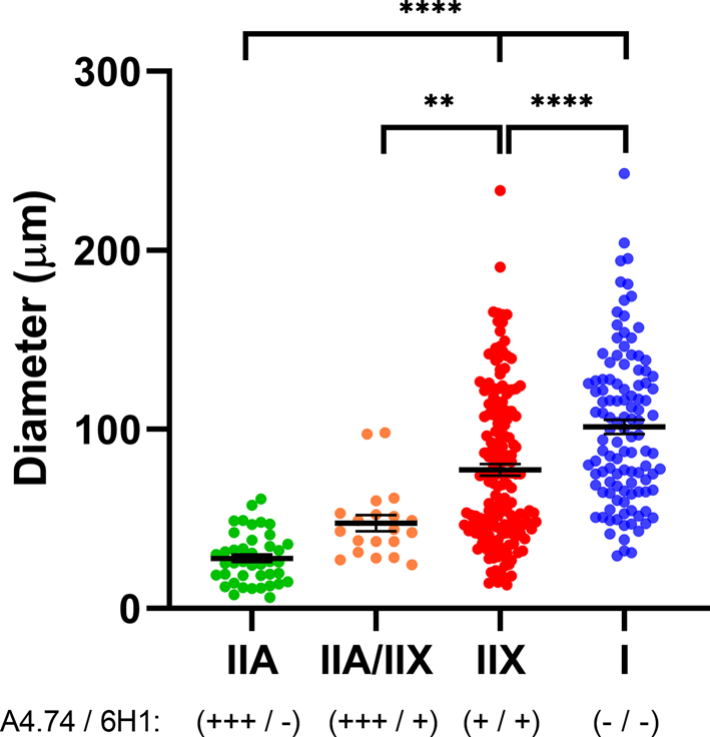
Fiber size diameters of immunohistochemical fiber types were measured in eight confocal fields of view at 10 × magnification. Fiber types were identified using a combination of staining patterns of the A4.74 and 6H1 antibodies [16]. Fibers were determined to be type MyHC-IIA with intense A4.74 staining and without 6H1 colocalization (n = 41), or MyHC-IIA/IIX hybrid fibers with intensive A4.74 positivity colocalizing with 6H1 staining (n = 20). MyHC-IIA and MyHC-IIA/IIX fibers were significantly smaller in diameter and more infrequent than MyHC-IIX fibers (n = 172) (weak A4.74 positivity colocalizing with 6H1 staining) and type I fibers (n = 112), with negative A4.74 and 6H1 staining but visualized and measured in the DAPI channel. Statistical significance as ** *p* < 0.01, ****p < 0.0001 was calculated by one-way ANOVA with Tukey’s multiple comparisons testing

High magnification (Fig. 3a) showed the filamentous appearance of the large inclusions and nemaline rods more clearly, while serial sections (Fig. 3b–f) confirmed that the filamentous tangles and associated nemaline rods occurred in type 2A fibers. Approximatively 1 to 9 type 2A fibers were present in each fascicle, with most fascicles harboring 4–5 type 2A fibers (as adjudged by combined findings in ATPase stains at pH 4.3, 4.6 and 9.4), but the estimated number of pure 2A fibers was complicated by the presence of fibers of poorly differentiated histochemical type. Immunocytochemical studies indeed showed presence of hybrid fibers co-expressing myosin IIX and IIA (Fig. 4a–d). Thus, hybrid fibers may also contain tangles. To better demonstrate the atrophy of type 2A fibers, we measured fiber size in sections immunoreacted for myosin IIA and IIX [16]. MyHC-IIA fibers had a mean fiber diameter of 28.0 ± 2.2 µm (Fig. 5) (mean ± SEM) with rare fibers as large as 61 µm. The mean diameter of hybrid MyHC-IIA/IIX fibers was 47.7 ± 4.5 µm with extremely rare fibers of up to 98 µm. MyHC-IIA and hybrid MyHC-IIA/IIX diameters were not significantly different (*p* = 0.254). MyHC-IIX fiber types had a mean diameter of 77.4 ± 3.2 µm (range:13–233 μm), while type 1 fibers had a mean diameter of 101.3 ± 4.0 µm (range: 30–243 µm). MyHC-IIA and MyHC-IIA/IIX fibers were less frequent and statistically smaller than pure MyHC-IIX and type I fibers.

**Fig. 6:**
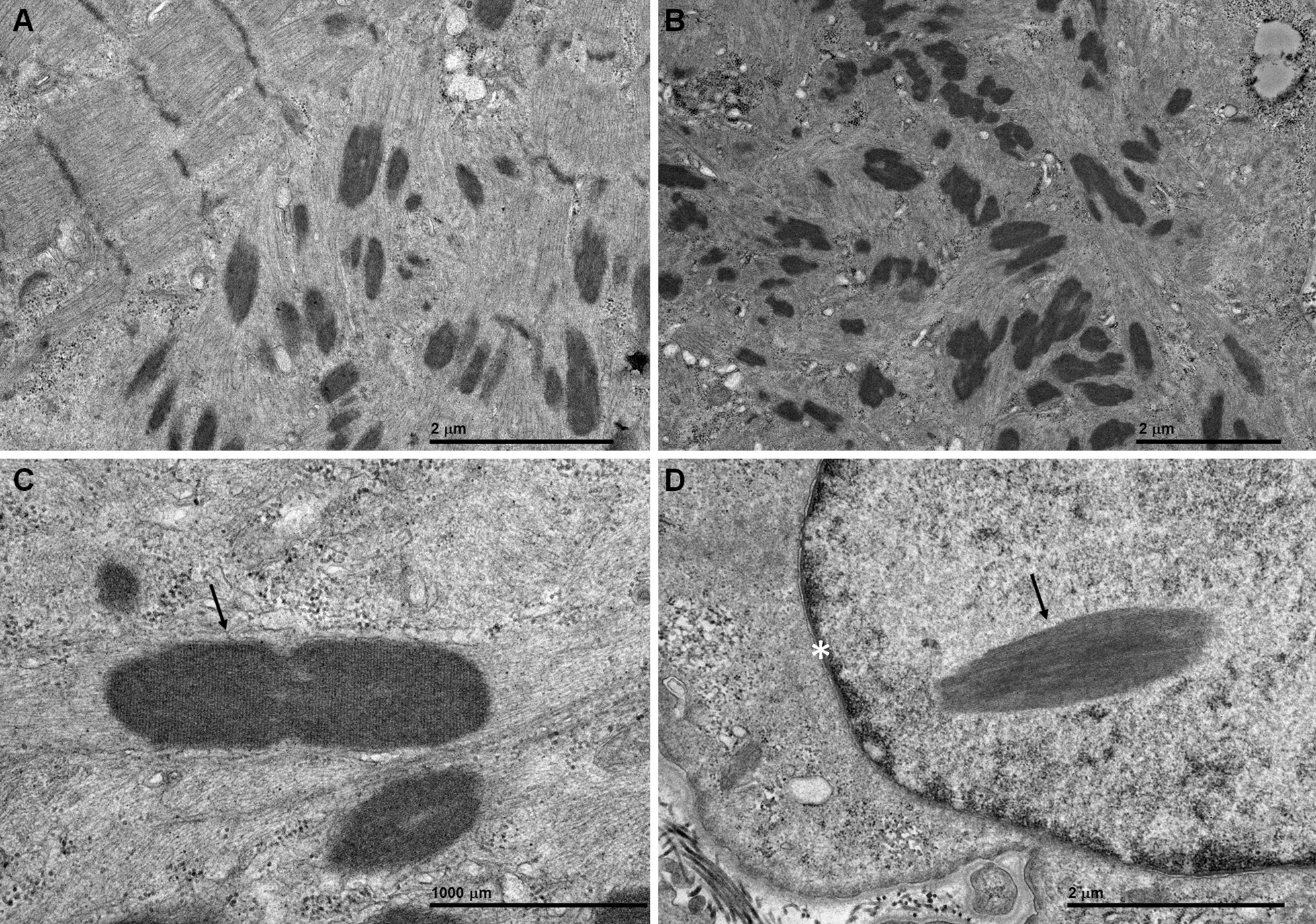
Ultrastructural findings. **a** Preserved sarcomeres in the left upper corner adjacent to an area of chaotically distributed myofibrils and clusters of nemaline rods (dark structures) in the right lower section, shown also in another fiber (**b**). **c** A classic nemaline rod (arrow) emanating myofilaments. **d** A nemaline rod (arrow) in a nucleus (asterisk indicates nuclear membrane). *Magnification: A, 25kx; B, 12kx; C, 60kx; D, 30kx*

Electron microscopy (EM) studies were performed on previously frozen muscle biopsy tissue. Tissue samples were taken directly from the − 80 °C freezer and separated into approximately 2 mm pieces. Individual pieces were quickly placed in ice-cold Trump’s fixative, making sure they remained frozen before placing into fixative. After approximately two hours, tissue was transferred to a 4 °C refrigerator and allowed to fix overnight. Following fixation, the tissue was washed with phosphate buffer, post-fixed in 1% osmium tetroxide, washed in H_2_O, dehydrated through a graded series of ethanol and acetone, and embedded in epon-araldite resin. Following a 24-h polymerization in a 60 °C oven, 0.1 µM ultrathin sections were prepared and then post stained with lead citrate and uranyl acetate. Electron micrographs were acquired using a JEOL 1400 Plus transmission electron microscope (JEOL, Inc., Peabody, MA) at 80 kV equipped with a Gatan Orius camera (Gatan, Inc., Warrendale, PA). The EM images (Fig. 6a–c) showed large areas of disorganized myofibrils oriented in multiple planes of direction, often trapping many nemaline rods, corresponding to the large tangles with rods seen on light microscopy. Occasionally nemaline rods were observed in nuclei (Fig. 6d).

### Genomic sequencing

Whole exome next generation sequencing, performed in a commercial laboratory (GeneDx, Gaithersburg, Maryland, USA) identified two heterozygous variants in *MYH2*. The first *MYH2* variant, c.3331C>T, p.Gln1111*, is predicted to result in truncation of the proximal tail region of the MyHC-IIA protein and is therefore predicted to be pathogenic. This variant has an extremely low allele frequency (3.98e−6, 1/251326) in large genetic databases (gnomAD) and shows possibly damaging (PolyPhen-2), damaging (SIFT, FATHMM, and fathmm-MKL), deleterious (LRT), disease causing (MutationTaster) effect through in silico analysis with a Combined Annotation-Dependent Depletion (CADD) score of 39. The second *MYH2* variant, c.1546T>G, p.Phe516Val, affects a highly conserved amino acid within the highly conserved catalytic motor head relay loop. *MYH2* c.1546T>G, p.Phe516Val is reported to have a very low allele frequency of 0.00000398 in large genetic databases (gnomAD, 1000 Genomes, and Exome Variant Server [EVS]), and shows probably damaging (PolyPhen-2), damaging (SIFT, FATHMM, and fathmm-MKL), unknown (LRT), and disease-causing (MutationTaster) effect through in silico analysis, with a Combined Annotation-Dependent Depletion (CADD) score of 25. Targeted Sanger sequencing showed that patient’s asymptomatic mother carries the c.1546T>G, p.Phe516Val variant, suggesting that the c.3331C>T, p.Gln1111* either arose from the asymptomatic deceased father or is *de novo*. The patient was also found to carry two heterozygous variants of unknown significance in *MYO9A* (c.5620A>C, p.Lys1874Gln and c.1148C>T, p.Ser383Phe), both detected also in the asymptomatic mother. No pathogenic or potentially pathogenic variants were detected in other genes so far known to cause neuromuscular diseases.

### Western blot

**Fig. 7:**
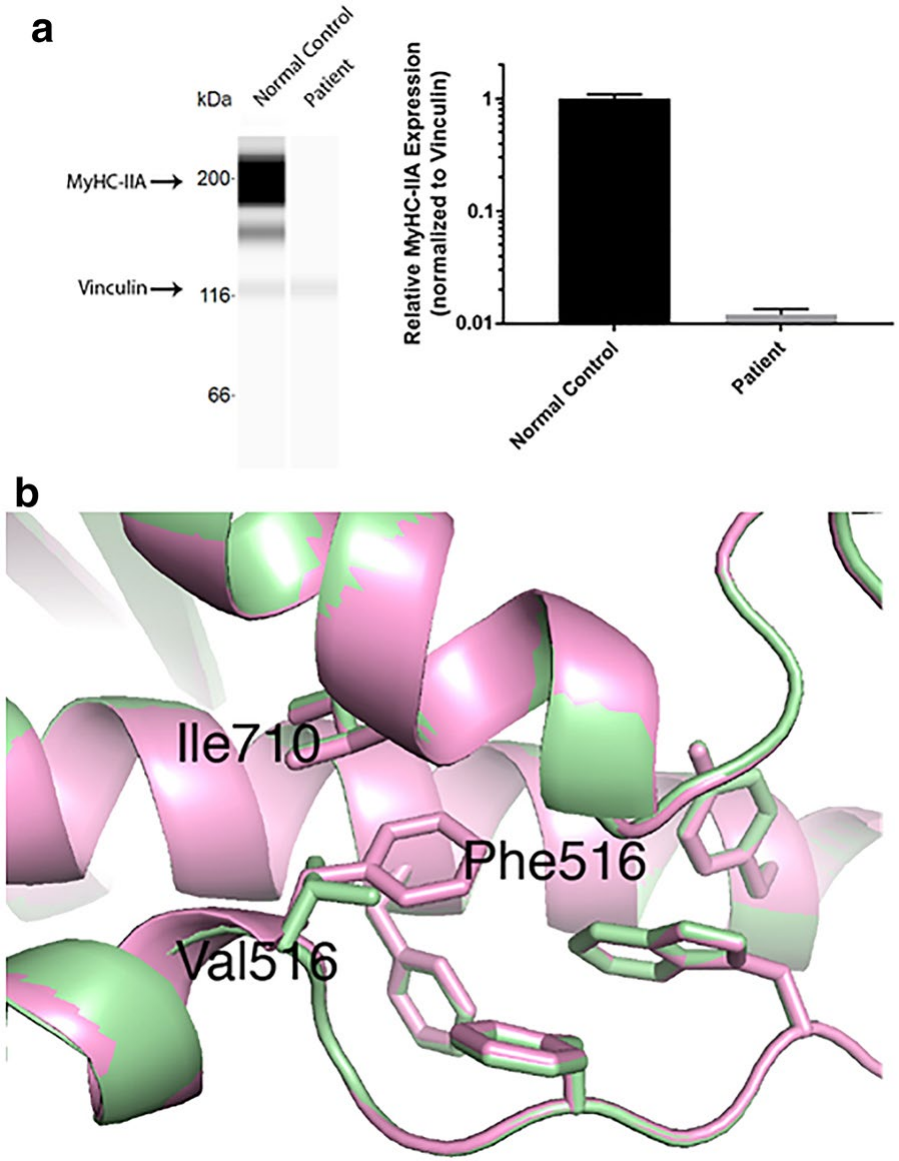
**a** Relative expression of MyHC-IIA protein in patient and age-matched control muscle tissue by Western blotting, showing marked reduction in MyHC-IIA in the patient. **b** Protein structure modeling displays the hydrophobic cavity region within the relay loop domain. In the wildtype protein, the phenylalanine at position 516 is in direct contact with an isoleucine at position 710. This interaction contributes to compaction of the hydrophobic region. The introduction of a valine at position 516 disrupts the central hydrophobicity of the domain and widens the cavity in relationship to the adjacent isoleucine. The 3D structures of wildtype and p.Phe516Val MyHC-IIA were modeled by homology using SWISS-MODEL [34] with the 2.3 Å resolution X-ray structure of *Bos taurus* cardiac myosin (Protein Data Bank entry 6FSA) [21] as template

Western blot analysis for MyHC-IIA protein concentrations were performed on total protein isolated from the proband patient’s muscle biopsy and muscle from an age-matched normal control subject. Protein lysates from 15 mg (dry weight) of muscle tissue were run at a concentration of 2.0 ug/ul per lane in quadriplicate using a 66–440 kDa separation module on a Wes Simple platform (ProteinSimple, San Jose, California, USA), according to the manufacturer’s protocol. MyHC-IIA protein bands were identified with mouse anti-human A4.74 antibody (1:10 dilution) as developed by Blau HM and colleagues [35] and obtained from the Developmental Studies Hybridoma Bank. MyHC-IIA band intensities were normalized to human vinculin protein (mouse anti-human antibody, 1:50 dilution) (R&D Systems, Minneapolis, Minnesota USA) in multiplexing analysis using the anti-mouse Luminol-S detection module and the Compass software (ProteinSimple). Mean MyHC-IIA protein illuminance in the affected patient sample, normalized to that of vinculin in each lane was reduced to 1.21 ± 0.15% (mean ± SEM) of that within the normal control sample (100.00 ± 9.15%) (Fig. 7a).

### MyHC-IIA protein structure–function modeling

Protein structure homology modeling using the X-ray structure of cow cardiac myosin as template predicts that the p.Phe516Val substitution in MyHC-IIA destabilizes the motor head relay loop domain. Phe516 is located in a hydrophobic cavity, where it directly contacts Ile710 (Fig. 7b). With a valine in place of a phenylalanine at position 516, the hydrophobic cavity is disrupted and the loop domain becomes less compact. As the relay loop domain acts as a fulcrum that regulates conformational change between high and low affinity states, this structural change may in turn affect the angular and rotational movements of the motor head converter, actin-binding region and ATP binding sites.

## Discussion and conclusions

Our patient carries two *MYH2* pathogenic variants leading to novel myopathologic findings featured by large filamentous tangles with clusters of nemaline rods and a classic clinical phenotype. One could hypothesize that the mutated MyHC-IIA might lead to inability to dimerize due to tail truncation and/or misfolded head region, resulting in the observed myofibril disorganization. The presence of nemaline rods in this patient with *MYH2*-myopathy is novel but not surprising, as rods are Z line-derived structures. In addition, rods can form not only in pure nemaline myopathy, but also in other muscle diseases caused by defects in sarcomeric and non-sarcomeric proteins, or as a result of metabolic derangements [24]. Of interest, nemaline rods can occur in normal extraocular muscles, in which most fibers stain for MyHC-IIA [10, 23]. Therefore, one could speculate that the mutated MyHC-IIA might exacerbate in limb skeletal muscle the physiological process of rod formation observed in normal extraocular muscles. The myopathological findings of our patient differ from the previous muscle pathologic descriptions in *MYH2*-myopathy which have included rimmed vacuoles, tiny p62 and ubiquitin positive inclusions or congophilic inclusions akin to inclusion body myopathy [28], minicore-like formations [6–9], but no nemaline rods. Previous ultrastructural studies have shown small cytoplasmic inclusions consisting of 15 and 20 nm tubulofilaments, Z-band streaming [1, 3, 19], disarrangement of sarcoplasmic myofilaments [13, 27], and nuclear filamentous inclusions [30]. Similarly to other *MYH2*-myopathy cases, which have shown selective reduction in the number and size of type 2A muscle fibers [2, 5, 13, 28], occasionally even leading to the lack of type 2A fibers [26], our patient showed paucity and atrophy of type 2A fibers, and severe reduction in MyHC-IIA expression. The coexistent nonspecific chronic myopathic changes, also present in our patient’s muscle biopsy, are not novel, except for the ring fibers. Indeed, some patients with *MYH2*-myopathy have shown dystrophic myopathological features [28].

Pathogenic *MYH2* variants have tended to cluster either within key functional domains in the motor head, or in the coiled-coil tail region as truncations [5]. Here we describe an additional two *MYH2* variants within the catalytic head and tail domains, being only the second report [30] of variants spanning both regions simultaneously. Although we cannot confirm that the patient’s *MYH2* variants are *in* *trans* (the patient’s father is deceased and sibling DNA is not available), the lack of infantile contractures, the late-pediatric onset of muscle weakness, the autosomal recessive nature of the previously reported *MYH2* variants leading to truncated protein, and the asymptomatic mother carrying the *MYH2* missense variant, all favor the compound heterozygous state of the two *MYH2* variants in the patient.

The p.Phe516Val variant occurs within the relay loop domain, a second configuration hinge adjacent to the SH1 domain. The relay loop domain harbors a highly conserved series of hydrophobic amino acids forming a cavity, about which the rotational angulation between the motor head converter, actin-binding region and ATP binding sites are coordinated. Our protein structure modeling suggests that the substitution of valine for phenylalanine at position 516, particularly in its direct relationship to an isoleucine residue at position 710, destabilizes that hydrophobic cavity. In the wildtype protein, the Phe516 to Ile710 interaction importantly contributes to domain compaction. In vitro studies of dominant negative inhibition of relay loop phenylalanine caused complete disruption of the lever arm motor activity by increasing the affinity to actin in the presence of ATP by > 100 fold [32]. The relay loop, also termed the activation loop, may therefore regulate MyHC-IIA ATPase activity between low and high actin affinity states, through conformational shifts linking the converter and lever arms [33]. In another case study, a homozygous recessive c.1498G>T, p.Glu500* produced bi-allelic protein truncation within the relay loop domain in a 48 year old with mild hyperCKemia, adolescence-onset neck flexors and symmetric limb girdle muscle weakness [6]. This patient also had early onset cataracts, in addition to the usual ophthalmoparesis.

Pathogenic autosomal recessive *MYH2* variants have been shown to occur in other critical globular head domains (Table 1), including the SH1 helix domain [3, 14, 15, 29], ATP binding Switch I [5, 26], the ATP binding P-Loop domain [26], the IQ calmodulin binding motif for myosin light chain interactions [13, 26, 30, 31], and the IQ motif with the actin binding domain [27]. Several recessive splice site changes produce exon skipping mutations in intervening sequences that scaffold the steric positioning of domains involving the actin binding site [27, 36]. Additional homozygous changes similarly disrupt intervening sequences between the ATP and actin domains [26].

The second allelic change in our patient, c.3331C>T, p.Gln1111*, occurred within the coiled-coil tail region. As p.Gln1111* is a stop codon mutation, its RNA product would result in a truncated transcript, which is commonly subjected to early degradation. Even it is translated into a protein product, this will be truncated, eliminating the carboxy-terminal domains that may be essential for titin, myomesin and M-protein binding within sarcomeric thick filaments [19]. This variant is also expected to remove the assembly competence domain, which is necessary for the intertwining of the heavy chain tail regions and MyHC-IIA dimerization. Although one cannot entirely exclude that our patient’s p.Gln1111* might be the only determinant of his phenotype (should this variant have risen *de novo* in the patient and his father was asymptomatic), on the basis of the current knowledge, it is unlikely that this single variant by itself is the only cause of the myopathy. Tail truncations have been reported in the literature as autosomal recessive traits, including a frame shift variant [26] in a neonatal presentation of marked ptosis, near complete ophthalmoplegia and mild proximal limb weakness, and in joint truncations of the head and tail [30].

Other mechanisms have included autosomal dominant missense point mutation disruptions of the ‘d’ position in alpha helix formation within the assembly competence domain, required for distal carboxy-terminal tail–tail intercalation and MyHC-IIA dimerization. One phenotype [2] associated with severe dysphagia, facial weakness, and hypotonia in infancy, while the second [1] occurred in a 20 year old with proximal leg, intrinsic hand weakness, ophthalmoplegia by age 25, prominent dysphagia and facial weakness suggestive of a phenotype overlapping with oculopharyngodistal myopathy spectrum.

The two *MYO9A* heterozygous missense variants, which were also detected in the patient, are of no clinical relevance as they were also present in the asymptomatic mother. In addition, pathogenic variants in *MYO9*, which encodes an unconventional myosin important for neuronal growth, have been associated with congenital myasthenic syndrome [18], but our patient had no electrophysiological features of a defect of neuromuscular transmission.

In conclusion, we present a pathogenic *MYH2* genotype producing loss of MyHC-IIA and novel muscle pathologic findings consisting of filamentous tangles with clusters of nemaline rods, broadening the currently known phenotype of *MYH2*-myopathy.

## Data Availability

The datasets used and/or analyzed during the current study are available from the corresponding author on reasonable request.

## Abbreviations

*MYH*: Myosin heavy chain (gene)
MyHC: Myosin heavy chain (protein)
c: Coding DNA
C: Cytosine
T: Thymine
p: Protein
Gln: Glutamine
*: Stop codon
G: Guanine
Phe: Phenylalanine
Val: Valine
ATP: Adenosine triphosphate
MRC: Medical Research Council
CK: Creatine kinase
Hz: Hertz
NCAM: Neural cell adhesion molecule
DSHB: Developmental Studies Hybridoma Bank
NADH: Nicotinamide adenine dinucleotide H+
SEM: Standard error of the mean
EVS: Exome variant server
Polyphen: Polymorphism phenotyping
SIFT: Sorting intolerant from tolerant
FATHMM (-MKL): Functional Analysis through Hidden Markov Models (-MKL algorithm)
LRT: Likelihood ratio test
CADD: Combined annotation-dependent depletion
Lys: Lysine
Ser: Serine
Ile: Isoleucine
SH1: Src homology 1
IQ: Isoleucine-glutamine
EM: Electron microscopy
DAPI: 4′,6-Diamidino-2-phenylindole
